# African Swine Fever Virus R238L and R298L Disrupt Lung Cell Collagen Formation and Cell Adhesion Pathway by Targeting Transcription Factors Containing zf-C2H2 Domain

**DOI:** 10.3390/vetsci13030236

**Published:** 2026-02-28

**Authors:** Siqi Niu, Fanghong Zhang, Jingchun Wen, Yiyun Wang, Alegria Agostinho Francisco, Beneque Alberto Anzol, Min Yao, Guoping Liu, Jianwu Wang, Tinghua Huang

**Affiliations:** 1 College of Animal Science and Technology, Yangtze University, Jingzhou 434025, China; siqi168niu@163.com (S.N.); fanghongzhang9388@163.com (F.Z.); jingchunwen21@163.com (J.W.); yiyun818wang@163.com (Y.W.); minyao@yangtzeu.edu.cn (M.Y.); 2 Angola Ministry of Agriculture Animal Husbandry and Veterinary Research Institute, Luanda 5682, Angola; alegria_be@yahoo.com.br (A.A.F.); beneque_al@yahoo.com.br (B.A.A.); 3 College of Agriculture, Yangtze University, Jingzhou 434025, China

**Keywords:** African swine fever virus, virus–host interaction, transcriptional regulation, extracellular matrix, transcription factors

## Abstract

The regulatory mechanisms of collagen formation and cell adhesion pathways during African Swine Fever Virus (ASFV) infection remain poorly understood. This study aims to investigate whether ASFV manipulates these pathways by targeting host transcriptional regulators. Through weighted Kendall correlation analysis of transcription factor binding sites (TFBSs) in differentially expressed genes (DEGs) from the lung tissue of ASFV-recovered pigs, we identified SP2 and KLF6 as key transcription factors (TFs) associated with collagen synthesis and cell adhesion, respectively. Domain–domain interaction prediction, followed by Förster resonance energy transfer (FRET) assays, confirmed that the ASFV proteins R238L and R298L directly bind to the zf-C2H2 domains of SP2 and KLF6. Furthermore, overexpression of R238L and R298L in HeLa and 3D4/21 cells significantly downregulated SP2 and KLF6 target genes involved in these pathways. Our findings reveal a novel mechanism by which ASFV proteins R238L and R298L interfere with host transcription factors SP2 and KLF6, potentially disrupting collagen matrix integrity and cell adhesion to facilitate viral pathogenesis.

## Simple Summary

Collagen formation (CF) and cell adhesion (CH) are essential biological processes that maintain cellular structure and communication. This study aimed to investigate whether and how the African Swine Fever Virus (ASFV) affects these host pathways. We identified two host transcription factors, SP2 and KLF6, which regulate the transcription of CF and CH genes. Furthermore, we found that two viral proteins, R238L and R298L, can bind directly to SP2 and KLF6 and reduce the transcription of their target genes.

## 1. Introduction

The focal adhesion (FA) pathway serves as a central signaling hub for cell–extracellular matrix (ECM) interactions. Multiple viruses hijack or modulate the FA pathway to facilitate viral entry, intracellular transport, replication, and release. It has been reported that adenovirus mediates invasion through binding to integrin αvβ3/αvβ5 in the FA pathway by its fibronectin [1]. Human papillomavirus initiates endocytosis via integrin α6β4 [2]. The spike protein of SARS-CoV-2 may enhance infection by interacting with integrin α5β1 [3]. Hepatitis C virus promotes internalization via integrin α6β4-FAK signaling [4]. Some viruses rely on FA signaling to establish replication compartments. For example, Enterovirus 71 infection activates the FAK-PI3K pathway, driving actin reorganization to form vesicle-like structures that support viral RNA replication [5]. Influenza virus utilizes FAK-RhoA signaling to promote intracellular transport of viral ribonucleoprotein [6]. Herpes simplex virus enhances movement from infected cells to neighboring cells via FAK-Rac1 signaling [7]. Epstein–Barr virus activates the integrin-FAK signaling pathway through its LMP1 protein to promote tumor cell invasion [8]. The FA pathway serves as a critical platform for successful viral infection of host cells.

Viruses can also affect collagen metabolism, leading to fibrosis (excessive deposition) or tissue fragility (insufficient deposition) [9]. Viruses, such as human cytomegalovirus, Epstein–Barr virus, hepatitis C virus, and human papillomavirus, can directly infect fibroblasts or mesenchymal stem cells. Following infection, viral-encoded proteins, such as HPV E6/E7, upregulate the expression of pro-fibrotic factors (e.g., TGF-β1, CTGF) and directly activate the transcription of collagen genes (e.g., COL1A1, COL3A1) [10,11]. Influenza, COVID-19, and dengue fever virus trigger intense release of pro-inflammatory cytokines (IL-1β, IL-6, TNF-α), which directly stimulate fibroblast proliferation and collagen synthesis [12]. The altered extracellular matrix forms a dynamic microenvironment that actively influences both the virus and the host [13]. Acting as a physical and signaling barrier, it physically restricts the diffusion of viral particles and may also impede the access of neutralizing antibodies or antiviral drugs to the infected cells [14].

ASFV is a large DNA virus with a complex double-layered capsid structure. The R238L gene it encodes plays a crucial role in the viral replication cycle. Located within the central conserved region of the ASFV genome, the R238L gene encodes a 238-residue enzyme for polyprotein processing. This enzyme is responsible for the processing of viral proteins through the cleavage of their precursors, including the maturation of at least five viral polyprotein precursors: p62, p220, p60, CP2475, and P1215R, constituting the rate-limiting step in viral particle maturation [15]. R238L gene deletion strains constructed via homologous recombination exhibit complete replication defects, failing to produce infectious viral particles in porcine alveolar macrophages [16].

The R298L gene of ASFV is a highly conserved, early-expressed gene (detected 2~4 h post infection). Located within the multigene family 505 region of the ASFV genome, R298L is identified as a non-essential, yet critical, virulence factor through gene knockout studies, with its deletion significantly impairing the virus’s pathogenicity in vivo [17]. The R298L protein features a short N-terminal cytoplasmic domain, a single hydrophobic transmembrane α-helix, and a large C-terminal luminal domain. It anchors to membrane structures within endomembrane systems, such as the endoplasmic reticulum (ER) and Golgi apparatus, and colocalizes with the marker proteins of these organelles [18]. The R298L protein acts as an inhibitor of the host innate immune system: (i) It suppresses the cGAS-STING signaling pathway, a core mechanism for the cellular recognition of cytoplasmic viral DNA and initiation of the type I interferon (IFN-I) antiviral response. The R298L protein specifically interacts directly with STING, a key host cell adapter protein [17]; (ii) It anchors STING to the endoplasmic reticulum membrane via its transmembrane domain, physically preventing STING transport from the ER to the Golgi apparatus/ER–Golgi intermediate compartment [19]; and (iii) The R298L protein’s “trapping” effect on STING effectively blocks the cGAS-STING pathway-dependent IFN-I production triggered by viral DNA, creating an “immunologically silent” microenvironment conducive to viral replication. Compared to wild-type virulent strains, ASFV strains lacking the R298L gene exhibit significantly reduced pathogenicity in domestic pigs. Infected pigs typically display only mild or no clinical symptoms, exhibit low viremia levels, and can survive while generating protective immune responses [20].

Although collagen formation and cell adhesion pathways are known to play critical roles in virus–host interactions, the regulatory mechanisms of these pathways during African swine fever virus (ASFV) infection remain largely unexplored. In particular, it is unclear whether ASFV modulates host transcriptional programs to disrupt these processes. Therefore, the objective of this study was to investigate whether ASFV interferes with collagen formation and cell adhesion pathways by targeting host transcription factors, and to identify the viral proteins involved in this regulatory mechanism.

## 2. Materials and Methods

### 2.1. Experimental Materials

The lung tissue samples utilized in this study were collected from a commercial pig farm in Jingzhou, China (30°32′ N, 112°23′ E), where an ASFV outbreak was first detected in August 2019 [21]. The farm is located in a subtropical monsoon climate zone, with average temperatures ranging from 28 to 32 °C and high humidity during the sampling period. All animals were clinically healthy Duroc × Large White × Landrace crossbred pigs, approximately 6 months of age, weighing between 80 and 100 kg, with no clinical signs of ASFV infection. Following the implementation of PCR detection and p72 antibody assay (ASFV p72 Antibody ELISA Kit, IDEXX Laboratories, Westbrook, ME, USA), we selected three groups of animals, with three individuals each, for sample collection: (i) p72-antibody-negative animals that were PCR-negative for ASFV, ELISA-negative for p72 antibody, and exhibited no symptoms of infection; (ii) low-p72-antibody animals that were PCR-negative for ASFV and low levels for p72-antibody (25% percentile); and (iii) high-p72-antibody animals that were PCR-negative for ASFV and high levels of p72-antibody (95% percentile). The sampling procedures carried out in this study complied with the Regulations for the Administration of Experimental Animals, as established by the Science and Technology Commission of China (No. 2006-398). Ethical approval for all animal-related protocols was granted by the Animal Ethics Commission of Yangtze University (Approval No. 2023-036; issued on 1 April 2023, in Jingzhou, Hubei, China). Prior to tissue collection, piglets were sedated through intramuscular administration of a tiletamine-zolazepam mixture (5 mg/kg, sourced from Zoetis Inc., Parsippany, NJ, USA) along with xylazine (2 mg/kg, obtained from Bayer AG, Leverkusen, Germany). Once unconsciousness was induced, euthanasia was completed via an intravenous overdose of sodium pentobarbital (100 mg/kg, supplied by Sigma-Aldrich, St. Louis, MO, USA). Cessation of vital signs, including the absence of a corneal reflex and the halting of respiration and heartbeat, confirmed death. Immediately thereafter, submandibular lung tissue specimens were carefully harvested using sterile surgical tools. These samples were subsequently divided into aliquots, rapidly frozen in liquid nitrogen, and preserved at −80 °C for later molecular analyses.

### 2.2. High Throughput Sequencing

The samples were shipped to the designated DNA facility on dry ice for subsequent RNA-seq analysis. Library preparation was performed according to the protocol outlined in the Illumina Truseq RNA sample preparation kit (Illumina Inc., San Diego, CA, USA). For each specimen, approximately 10 μg of total RNA was employed for library construction and RNA sequencing. Sequencing was executed on an Illumina HiSeq 2500 platform (Illumina Inc., USA) utilizing a single-read 50 bp sequencing mode. Following sequencing, raw reads were subjected to a data filtration process in order to eliminate low-quality sequences and obtain clean, high-quality reads, in accordance with the manufacturer’s guidelines. For alignment of clean reads to the reference genome, the software Hisat2 [22] (version 2.2, accessed March 2024) was utilized. The reference genome sequence (Sus Scrofa 11.1, accessed March 2024) was retrieved from the NCBI genome database [23]. To quantify gene expression levels, read counts per gene were first calculated using HTSeq-count [24] (accessed March 2024), enabling comparative analysis of expression profiles across samples. Library normalization was subsequently carried out via the median of ratios method. For the identification of differentially expressed genes, the DESeq2 R package [25] was applied, with significance defined by a false discovery rate (FDR) ≤ 0.05 and a fold change (FC) either ≥1.5 or ≤0.67. Functional enrichment analysis of the identified genes was then performed using the DAVID annotation tool (accessed May 2024). The datasets supporting the findings of this study have been made publicly available in the NCBI GEO database under the accession code GSE316979.

### 2.3. Identification of Key Transcription Factors

The identification of the key TFs that regulate the differentially expressed genes was achieved through a Kendall’s tau rank correlation analysis of the potential of TF binding and the extent of the differential expression of the target genes of TFs utilizing the FLAVER v2.0 webserver (accessed April 2024) developed by Yao and Huang et al. [25,26,27]. The analysis emphasizes genes with larger weights while de-emphasizing genes with smaller weights. As Shieh’s research indicates, the weighted Kendall’s τ assumes the form of Equation (1). The limiting distribution (LD) can be derived from Equation (2). As the value of *n* approaches infinity, the LD value approaches N(0, 1). Thus, the value of the *p*-value can be estimated:

(1) $$\tau_{w}=2/\left[{\left(\sum_{i}^{n}v_{i}\right)}^{2}-\sum_{i}^{n}v_{i}^{2}\right]\cdot \sum_{i>j}^{n}v_{i}v_{j}sgn\left(i-j\right)sgn\left(R_{i}-R_{j}\right)$$

If X <, =, or >0, then sgn (X) = −1, 0, or 1. The v_i_ denotes the weighting function, which is bounded by [1, n] and ranges from 0 to 1:

(2) $$LD=\sqrt{n}\tau_{w}\frac{3\underset{n\to \infty }{\lim}n^{-1}\sum_{x}^{n}v_{x}}{2\sqrt{\underset{n\to \infty }{\lim}n^{-1}\sum_{x}^{n}v_{x}^{2}}}$$

### 2.4. Prediction of African Swine Fever Virus–Host Interactions Based on the Domain–Domain Approach

Protein sequence data and annotation information for ASFV, as well as protein sequence data and annotation information for pigs, were downloaded from the NCBI Genome database. Domain–domain interaction data were obtained from the PPIDM database (accessed April 2024) [28]. The prediction followed the Cristian method [29] through these steps: (i) extracting motif data from Pfam corresponding to PPIDM; (ii) aligning extracted motifs with ASFV protein sequences to establish ASFV–Motif1 pairs; (iii) aligning extracted motif sequences with pig protein sequences to establish pig–Motif2 pairs; and (iv) constructing the ASF–Motif1–Motif2–pig regulatory network based on Motif1–Motif2 interactions, where Motif1–Motif2 information originated from the PPIDM database. The e-value for ASFV–Motif1 was controlled below 1 × 10^−6^, encompassing approximately 45% of ASFV proteins. The e-value for Motif2–pig protein interactions was controlled below 1 × 10^−6^, covering about 20% of pig proteins.

### 2.5. Construction of Eukaryotic Expression Vectors for African Swine Fever Virus Host Protein Domains

The DNA sequence encoding domain A (Pkinase, PK_Tyr, or Ank_2) and the SNAP, CLIP tag were synthesized by Sangon Biotech (Shanghai, China), wherein the domain A sequence was flanked by *EcoR* I and *Nhe* I restriction sites, and the SNAP tag was flanked by *Nhe* I and *Xho* I restriction sites. The synthetic DNA sequence for domain B (encoding zf-C2H2 from the host SP2 or KLF6 gene) contains *EcoR* I and *Nhe* I restriction sites at its 3′ and 5′ ends, respectively. The synthetic DNA sequence encoding the CLIP tag contains *Nhe* I and *Xho* I restriction sites at its 3′ and 5′ ends, respectively (detailed sequence information is provided in Supplementary File S1). The 3′-end of Domain A sequence and the 5′-end of the SNAP sequence form complementary sticky ends after digestion with *Nhe* I endonuclease. Similarly, the 3′-end of Domain B sequence and the 5′-end of the CLIP sequence produce complementary sticky ends after digestion with *Nhe* I endonuclease. The *Nhe* I-digested products of domain A and SNAP were ligated using T4 DNA ligase and cloned into the pcDNA3.1-Flag plasmid (210342) using *EcoR* I and *Xho* I restriction enzymes, yielding the pcDNA3.1-Pkinase-SNAP, pcDNA3.1-PK_Tyr-SNAP, and pcDNA3.1-Ank_2-SNAP plasmids. The *Nhe* I-digested products of domain sequence B and CLIP were ligated using T4 DNA ligase and cloned into the pcDNA3.1-Flag plasmid using *EcoR* I and *Xho* I restriction enzymes, yielding the pcDNA3.1_SP2-zf-C2H2-CLIP and pcDNA3.1_KLF6-zf-C2H2-CLIP plasmids. Using primers containing *EcoR* I and *Xho* I restriction sites, fragments encoding the R298L-Pkinase domain, R298L-PK_Tyr domain, and R238L-Ank_2 domain were amplified. After digestion with *EcoR* I and *Xho* I, the fragments were inserted into the linearized pcDNA3.1 vector, resulting in the recombinant plasmids pcDNA3.1-Pkinase, pcDNA3.1-PK_Tyr, and pcDNA3.1-Ank_2.

### 2.6. Cell Culture, Transfections, and Förster Resonance Energy Transfer (FRET) Assay

Porcine alveolar macrophage cell line 3D4/21 was cultured in DMEM supplemented with 10% fetal bovine serum (FBS), at 37 °C in a 5% CO_2_ humidified incubator [30]. Cells were seeded in 6-well plates at a density of 3 × 10^6^ cells per well. After allowing the cells to adhere for 24 h, plasmid transfection was performed when the cell confluence reached approximately 60%. Cells were transiently transfected using Lipo6000™ reagent, according to the manufacturer‘s instructions (C0526, Beyotime, Shangai, China). A total of 2.5 µg of DNA (plasmid A-SNAP:plasmid B-CLIP = 1:1) and 5 μL of Lipo6000™ reagent were separately mixed with 125 μL of DMEM medium (without antibiotics and serum). The DNA and Lipo6000™ were mixed and incubated for 15 min at room temperature to form transfection complexes, which were then added to 3D4/21 cells. After 4 h, the transfection complex media were removed, and the complete DMEM medium was added. The cells were incubated for 36 h at 37 °C, 5% CO_2_. The cells were labeled with 3 μM SNAP-Cell^®^ 430 (NEB, S9109S, Ipswich, MA, USA) and 5 μM CLIP-Cell™ 505 (NEB, S9217, Ipswich, MA, USA) in labeling media for 30 min at 37 °C, 5% CO_2_.

The cells labeled with SNAP-Cell^®^ 430 and CLIP-Cell™ 505 were collected by scraping and were washed twice with ice-cold PBS containing 1% FBS. Subsequently, the cells were resuspended in the same ice-cold buffer at a concentration of 1 × 10^6^ cells/mL for flow cytometer assay. Samples were analyzed on a BD LSRFortessa flow cytometer equipped with a 405 nm laser and the following detection channels: (i) the blue channel (470/40 nm filter) detects donor fluorescence; and (ii) the green channel (525/50 nm filter) detects FRET signals. Prior to acquisition, voltage calibration and fully automated compensation calculations were performed using single-stained samples (SC430 only, CC505 only) for each fluorescence channel to eliminate spectral overlap. Each sample group collected at least 10,000 live cell events. Raw data were analyzed using FlowJo 10.8 software. Fragments were first gated out using forward scatter (FSC-A) and side scatter (SSC-A), followed by gating on FSC-A vs. FSC-H to select single-cell populations for subsequent analysis. After applying the automatic compensation matrix, the derived parameter FRET ratio = Comp-430-A (FRET channel)/Comp-505-A (donor channel) was created. The average FRET ratio values were calculated for the experimental group and each control group. Statistical testing was performed using the R 4.2 ANOVA package. A *p*-value < 0.05 was considered statistically significant.

### 2.7. Real-Time PCR Assay

According to the method described in Section 2.6, the recombinant plasmids, pcDNA3.1-Pkinase, pcDNA3.1-PK_Tyr, and pcDNA3.1-Ank_2, were transfected into 3D4/21 cells and HeLa cells, respectively. As controls, siRNA-SP2 and siRNA-KLF6 were transfected separately into the cells (detailed sequence information of siRNA is provided in Supplementary File S1). Cells were harvested at 72 h post transfection; total RNA was subsequently extracted using the MiniBEST Universal RNA Extraction Kit (9769, Takara, Beijing, China). RNA concentration and purity were measured using a Nano-400A spectrophotometer (Wuhan Aivest Technology Co., Ltd., Wuhan, China), with an A260/A280 ratio between 1.9 and 2.1 considered; an RNA integrity number (RIN) greater than 9.0 was deemed acceptable. Complementary DNA (cDNA) was synthesized from the extracted RNA through reverse transcription employing the PrimeScript™ RT Reagent Kit (RR037B, Takara, Beijing, China). The primer sequences used for amplifying host transcription factors and their corresponding target genes are provided in Supplemental File S1. Quantitative real-time PCR assays were carried out with the TB Green^®^ Premix DimerEraser™ (RR091B, Takara, Beijing, China) strictly adhering to the manufacturer’s protocol. Relative expression levels of the target genes were determined using the 2−ΔΔCT method, with GAPDH serving as the internal reference for normalization.

## 3. Results

### 3.1. Lung Transcriptome Analysis of Individuals with Different States of Infection

A total of three groups of animals—p72-antibody negatives, low p72-antibody and high p72-antibody—with three animals per group were assayed using the RNA-seq technique. Analysis of the RNA-seq data revealed an average of 22 million reads per sample, leading to the identification of 16,717 distinct transcripts in total.Statistical analysis revealed that 169 transcripts exhibited significant differential expressions (FDR < 0.01 and FC > 2.0) between the high p72-antibody and p72-antibody negative animals. Comparison between animals with low p72-antibody levels and those testing negative revealed a total of 6936 transcripts that were significantly differentially expressed (FDR < 0.01, FC > 2.0). RNA-seq analysis revealed a total of 1525 transcripts exhibiting significant differential expression (FDR < 0.05) when comparing animals with high p72-antibody levels to those with low p72-antibody levels. The 30 most significantly modulated genes from this comparison are presented in Table 1. For the complete set of differentially expressed genes identified across all group comparisons, please refer to Supplemental Data S1.

A comparison between high and low p72-antibody animals revealed 40 inflammation-related DEGs, including chemokine receptors, TNF receptors, and interleukins. Additionally, 15 interferon-production-related DEGs were identified such as ABL1, CD2, DDX3X, and HSPD1. Furthermore, 28 apoptosis-related DEGs were identified, including ABL1, BCL2A, and FAIM. Three MHC I-associated DEGs, NLRC5, CIITA, and HSPH1, were also identified.

Notably, among the differentially expressed genes between high and low p72-antibody animals, we observed several collagen-related genes (such as COL4A2, COL4A6, and HSPG2) and cell adhesion-related genes (such as ITGA3, ITGB4, and CDH23) (Table 1). These findings prompted us to further investigate whether ASFV specifically targets transcription factors regulating collagen formation and cell adhesion pathways, which became the central focus of this study.

### 3.2. Identification of Transcription Factors Associated with the Differential Express Genes

**Figure 1:**
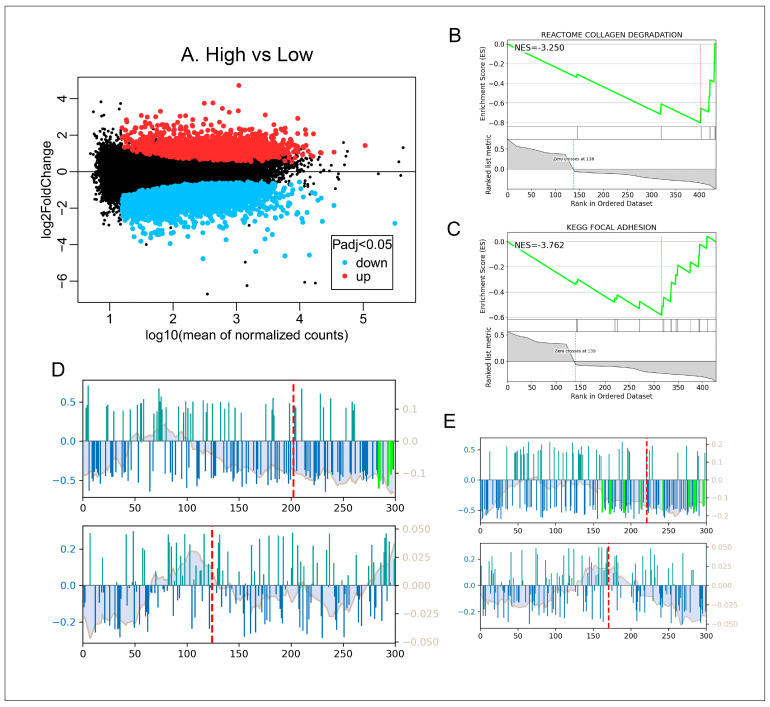
Discovery of key transcription factors (TFs) in porcine lung transcriptome data from animals with differing levels of titers of ASFV p72-specific antibodies: (**A**) plot of differentially expressed genes between high and low p72 antibody animals. Representative enriched pathway for SP2 and KLF6’ target genes were shown in (**B**,**C**), respectively.The correlation graphs for SP2 and KLF6, illustrating the relationship between their binding potential and the differential expression of their respective target genes, are shown in panels (**D**,**E**). The upper plot of (**D**,**E**) illustrates genes that have a TF binding site;. the lower plot depicts genes lacking TF binding sites, while the x-axis indicates the rank order of their differential expression levels. In this visualization, bar height serves as a quantitative measure of the binding potential between a TF and its target genes. Bars rendered with dark green color indicate the target gene was upregulated in the differentially expressed gene list while the dark blue color indicated downregulation. Bars highlighted with green represented the edge genes identified in the pathway enrichment analysis.

A total of 325 TFs were associated with the DEGs between the low p72-antibody and p72-antibody negative animals (FDR < 0.01). A total of 134 TFs were associated with the DEGs between the high p72-antibody and low p72-antibody animals (FDR < 0.01). For a comprehensive list of the TFs for differential genes compared in each group, refer to Supplemental Data S2. A correlation graph for two representative TFs, SP2 and KLF6, are shown in Figure 1D,E. SP2 and KLF6 were downregulated 6.3-fold and 3.87-fold (FDR < 0.001), respectively, between the high p72-antibody and low p72-antibody animals. Furthermore, candidate regulator TF SP2 was identified as being responsible for the regulation of 19 DEGs, which were associated with the COLLAGEN DOWREGULATION pathway. Meanwhile, regulator TF KLF6 was identified as regulating 17 DEGs in the FOCAL ADHESION pathway. It should be noted that these findings currently represent correlative associations between the transcription factors and the differentially expressed gene; causality has not yet been directly demonstrated at this stage. The downregulation of SP2 and KLF6, together with their target genes, suggested a potential role in ASFV infection that warranted further experimental validation, which is discussed in the following sections.

### 3.3. R238L and R298L Are Candidate ASFV Proteins Regulating Host Cell Adhesion and Collagen Pathways

Using the domain–domain method to search for the ASFV proteins acting on host’s TFs SP2 and KLF6 revealed that the domain located at residues 46 to 276 of ASFV’s R298L shared a similarity with the Pkinase domain (Evalue < 10^−3^). The host SP2 protein’s domain, located at residues 542 to 607, exhibits similarity to the zf-C2H2 domain (Evalue < 10^−3^). According to PPIDM database’s information, the Pkinase domain and zf-C2H2 domain exhibit strong interaction (rated silver). The ASFV’s R298L protein also exhibits high similarity (Evalue < 10^−3^) between its domain, located in residues 65 to 273, and the PK_Tyr domain, based on evidence of strong interactions between the PK_Tyr domain and zf-C2H2 domain in the PPIDM database. Additionally, the ASFV’s R238L protein exhibits high similarity (Evalue < 10^−3^) between residues 15 to 110 and the Ank_2 domain. Based on PPIDM database’s information, Ank_2 domains can also interact with zf-C2H2 domains (rated silver). The amino acid sequence of KLF6 also contains a zf-C2H2 domain (residues 219 to 316) which is highly similar with SP2; theoretically, this domain could also be recognized by the R298L’s Pkinase domain (residues 46 to 276), R298L’s PK_Tyr domain (residues 65 to 273), or the R238L’s Ank_2 domain (residues 15 to 110).

### 3.4. Construction of FRET Expression Vectors

**Figure 2:**
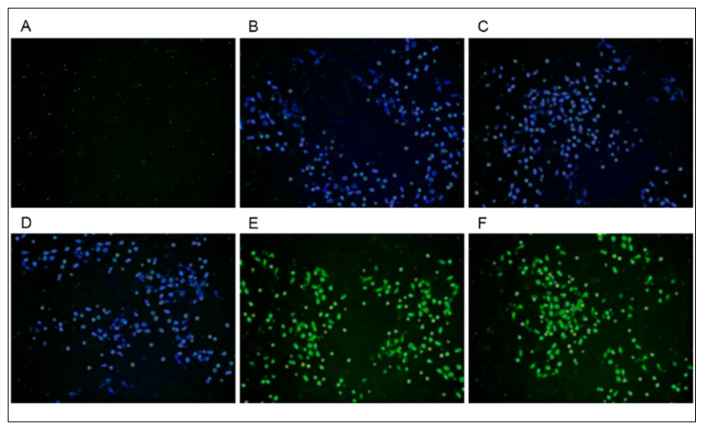
Testing the fluorescence signals of the cells transfected with fusion plasmid: plot (**A**) shows an 3D4/21 cell sample transfected with the control plasmid. Following transfection of the pcDNA3.1-Pkinase-SNAP (**B**), pcDNA3.1-PK_Tyr-SNAP (**C**), or pcDNA3.1-Ank_2-SNAP (**D**) plasmids, 3D4/21 cells were incubating with SNAP-Cell^®^ 430 (SC430) and fluorescence signals were observed under 405 nm excitation light. 3D4/21 cells transfected with pcDNA3.1-SP2-zf-C2H2-CLIP (**E**) or pcDNA3.1-KLF6-zf-C2H2-CLIP (**F**) plasmids were incubating with CLIP-Cell™ 505 (CC505) and fluorescence signals were observed under 488 nm excitation light.

In this study, we constructed five eukaryotic expression vectors: pcDNA3.1-Pkinase-SNAP; pcDNA3.1-PK_Tyr-SNAP; pcDNA3.1-Ank_2-SNAP; pcDNA3.1-SP2-zf-C2H2-CLIP; and pcDNA3.1-KLF6-zf-C2H2-CLIP. Among these, pcDNA3.1-Pkinase-SNAP fuses the Pkinase domain (residues 46 to 276) of the ASFV’s R298L protein with a SNAP-tag. pcDNA3.1-PK_Tyr-SNAP fuses the PK_Tyr domain (residues 65 to 272) of the ASFV’s R298L protein with a SNAP-tag. pcDNA3.1-Ank_2-SNAP fusion expresses the Ank_2 domain (residues 15–110) of the ASFV’s R238L protein and a SNAP-tag. Following transfection of the three vectors into 3D4/21 cells and incubating with SNAP-Cell^®^ 430 (SC430), distinct blue fluorescence signals were observed under 405 nm excitation light, confirming successful construction of the SNAP SC430 fusion vectors (Figure 2B,C). The pcDNA3.1-SP2-zf-C2H2-CLIP vector fused the zf-C2H2 domain (covering residues 542 to 607 of the porcine SP2 protein) and CLIP-tag. The pcDNA3.1-KLF6-zf-C2H2-CLIP vector fused the zf-C2H2 domain (covering residues 219 to 316 of the porcine KLF6 protein) with a CLIP-tag. Transfection of the aforementioned vectors into 3D4/21 cells and incubation with CLIP-Cell™ 505 (CC505) yielded distinct green fluorescence signals under 488 nm excitation light, confirming successful construction of the CLIP CC505 fusion vectors (Figure 2E,F).

### 3.5. FRET Flow Cytometry Detected Domain–Domain Interactions

**Figure 3:**
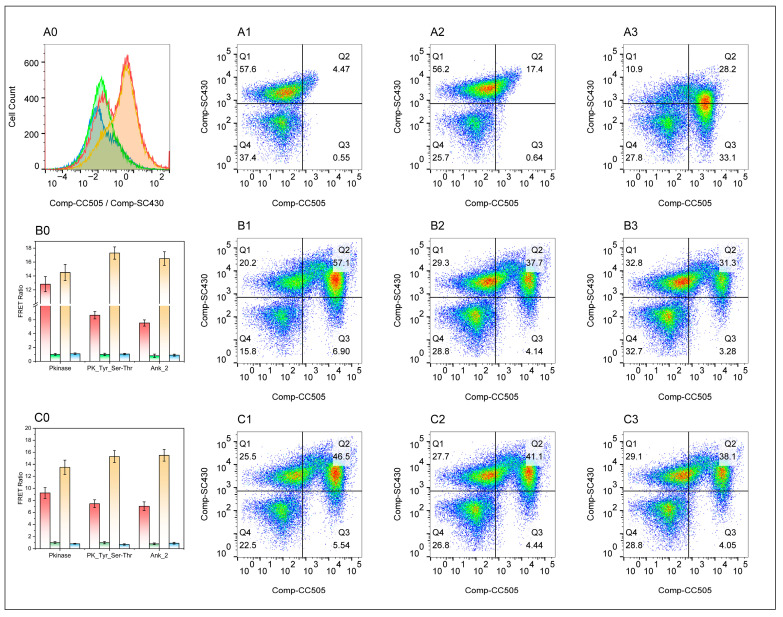
Representative dual-channel flow cytometry scatter plots illustrating the förster resonance energy transfer (FRET) assay results. Scatter plots displaying CC505 intensity on the X-axis and SC430 intensity on the Y-axis are shown in panels (**A1**–**A3**), with data presented for the negative control, SC430 only, and CC505 only groups, respectively. The results of cells co-transfected pcDNA3.1-SP2-zf-C2H2-CLIP with pcDNA3.1-Pkinase-SNAP, pcDNA3.1-PK_Tyr-SNAP, or pcDNA3.1-Ank_2-SNAP are presented in plot (**B1**–**B3**). The results of cells co-transfected pcDNA3.1-KLF6-zf-C2H2-CLIP with pcDNA3.1-Pkinase-SNAP, pcDNA3.1-PK_Tyr-SNAP, or pcDNA3.1-Ank_2-SNAP are presented in plot (**C1**–**C3**). A cross gate demarcates the two populations in plot (**A0**), with percentages provided for the SC430^−^/SC430^+^ and CC505^−^/CC505^+^ subsets. This panel also illustrates the compensated CC505/SC430 ratio distribution via histograms for the negative control (green), SC430 only (blue), CC505 only (red), and a representative field sample (yellow), where overlapping populations are indicated by shaded areas. The bar graph in plot (**B0**,**C0**) presented compensated CC505/SC430 ratios for negative control (green), the SC430 only (blue), the CC505 only (red), and filed sample (yellow) for Pkinase, PK_Tyr, and Ank_2 domain respectively.

Before compensation, SC430 monochromatically stained cells exhibited significant fluorescence bleed into the green channel (Figure S1A). After applying automatic compensation, the signal from this cell population in the green channel was effectively subtracted, demonstrating that SC430 achieves precise correction for cross interference in the green channel (Figure S1B). When the SC430 only sample was excited by the 405 nm laser, a strongly positive cell population appeared on the blue channel, representing the normal emission of SC430 upon excitation by its optimal light. In the green channel, a distinct but relatively weaker positive cell population also appeared. This signal originated from SC430’s “leakage” or “cross-interference”, as the tail of SC430’s emission spectrum spread into the detection range of the green channel. The SC430 only flow cytometry scatter plot shows the cell population exhibiting an upward dispersion along the Y-axis (blue channel) and pronounced rightward dispersion along the X-axis (green channel), forming a diagonal distribution sloping upward and to the right (Figure 3(A2) and Figure S1A). When the 405 nm laser was used to excite the CC505 only sample, the blue channel (detecting SC430) showed low signal intensity. This is because CC505’s optimal excitation wavelength is ~645 nm; its excitation efficiency under 405 nm laser light is very low. A distinct positive cell population appears in the green channel (detecting CC505) because light at a non-optimal wavelength (640 nm) partially excites CC505, with emission detected in the green channel (~670 nm). The scatter plot for CC505 only (405 nm excitation) shows the cell population tightly clustered near the background on the Y-axis (blue channel) and spreading to the right on the X-axis (green channel), forming an approximately vertical “short rod-shaped” distribution (Figure 3(A3)).

The flow cytometry results from the FRET experimental samples revealed a marked upward shift in cellular fluorescence signals. Concurrently, the overall distribution exhibited a significant rightward shift, forming a cell population that dispersed in a right-upward direction. Scatter plots of FRET experimental samples showed the cell population resembling a “fan opening upward and to the right” (Figure 3(B1–B3,C1–C3)). This demonstrates that, in the presence of SC430, CC505 emitted stronger fluorescence than when present alone, suggesting that energy transfer occurred. Overlaying the scatter plots of the CC505 only sample with those of the FRET sample revealed that the cell clusters in the FRET experimental sample were overall, and significantly higher in the green channel, compared to the CC505 only sample (Figure S1F). This provides the most direct visual evidence for FRET’s positivity.

Quantitative analysis revealed that in 3D4/21 cells co-transfected with three SNAP plasmid and pcDNA3.1-SP2-zf-C2H2-CLIP plasmids, respectively, the average FRET Ratio in the Pkinase group (12.82 ± 1.09) was 12-fold higher than that in the SC430 only group (0.95 ± 0.05) (*p* < 0.001) and 10-fold higher than that in the negative control group (NCG, 0.33 ± 0.02) (*p* < 0.01). These results indicate an efficient fluorescence resonance energy transfer occurred between SC430 (donor) and CC505 (acceptor) under these experimental conditions, suggesting that close-range interactions existed between the R238L Pkinase domain and the zf-C2H2 domain of SP2 within cells. The average FRET ratio in the PK_Tyr_Ser-Thr group (6.65 ± 0.53) was significantly higher than that in the SC430 only group (1.0 ± 0.18) and the NCG (1.05 ± 0.1) (*p* < 0.01). Quantitative analysis, as shown in Figure 3(B0), indicates that under these experimental conditions, FRET occurred between SC430 (donor) and CC505 (acceptor). This suggests that the PK_Tyr_Ser-Thr domain of R238L and the zf-C2H2 domain of SP2 undergo close-range interactions within cells. The average FRET ratio in the Ank_2 group (5.53 ± 0.45) was six times higher than that in the SC430 NCG (0.8 ± 0.25) (*p* < 0.01) and five times higher than that in the NCG (0.9 ± 0.14) (*p* < 0.0001). These results indicate that FRET between SC430 (donor) and CC505 (acceptor) occurred under experimental conditions, suggesting that close-range interactions existed between the Ank_2 domain of R298L and the zf-C2H2 domain of SP2 within cells. In cells co-transfected with three SNAP plasmids and pcDNA3.1-KLF6-zf-C2H2-CLIP plasmids, efficient FRAT occurred between SC430 (donor) and CC505 (acceptor), suggesting close-range interaction between the R238L Pkinase domain and the zf-C2H2 domain of KLF6 within cells (Figure 3(C0)).

It should be noted that the interactions characterized in this study were demonstrated using specific domains (the Pkinase, PK_Tyr, and Ank_2 domains of ASFV proteins R238L/R298L; the zf-C2H2 domains of SP2 and KLF6) rather than the full-length proteins. Furthermore, these assays were performed under plasmid-driven overexpression conditions in 3D4/21 cells. Therefore, while these results provide strong evidence for the physical proximity and potential interaction between these viral and host protein domains, they do not fully recapitulate the endogenous conditions during a natural ASFV infection. Future studies utilizing full-length proteins under physiological expression levels, such as during viral infection or through co-immunoprecipitation assays with endogenous proteins, are necessary to confirm the precise nature and functional consequences of these interactions in the context of the whole virus.

### 3.6. African Swine Fever Virus Protein Downregulates Host Gene Expression

**Figure 4:**
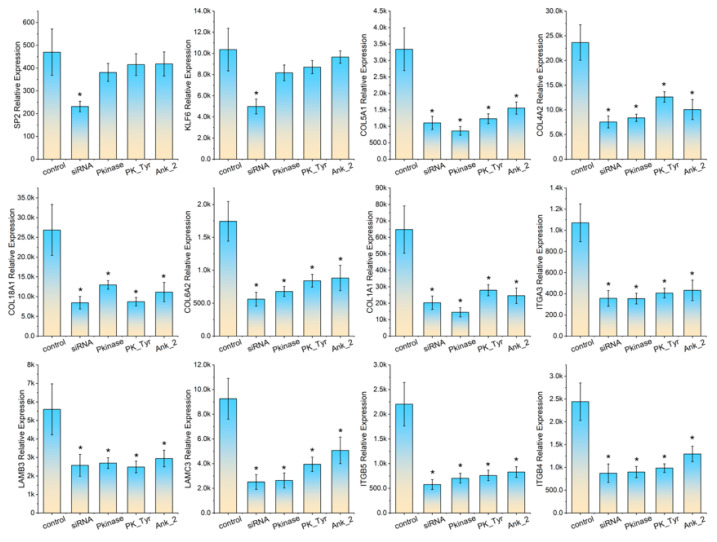
3D/421 cells were transfected with SP2 siRNA, KLF6 siRNA, pcDNA3.1-Pkinase, pcDNA3.1-Pkinase-PK_Tyr, or pcDNA3.1-Pkinase-Ank_2 plasmids; subsequently, the transcriptional levels of SP2, KLF6, and their target genes were measured. Bar heights indicate expression levels normalized to GAPDH, and asterisks mark statistically significant differences (*p* < 0.05).

We selected five KLF6 target genes (ITGA3, LAMB3, LAMC3, ITGB5, ITGB4) and five SP2 target genes (COL5A1, COL4A2, COL18A1, COL6A2, COL1A1) for real-time PCR validation. The total RNA extracted demonstrated good integrity and acceptable purity (RIN > 9.0), rendering the samples suitable for real-time PCR experiments. All primers passed melting curve testing, with no template control showing significant amplification signals (Ct values > 35). In cultured 3D4/21 cells, siRNA-mediated knockdown of SP2 and KLF6 significantly reduced their respective mRNA levels. Accordingly, SP2 knockdown downregulated the transcriptional levels of its target genes COL5A1, COL4A2, COL18A1, COL6A2, and COL1A1, while KLF6 knockdown similarly decreased the expression of its target genes ITGA3, LAMB3, LAMC3, ITGB5, and ITGB4. Following transfection of 3D4/21 cells with pcDNA3.1-Pkinase, pcDNA3.1-PK_Tyr, or pcDNA3.1-Ank_2 plasmids, no significant changes were observed in the mRNA levels of SP2 and KLF6 compared to the control group. However, the mRNA levels of target genes of both SP2 and KLF6 showed varying degrees of downregulation. Notably, transfection with pcDNA3.1-Pkinase and pcDNA3.1-PK_Tyr plasmids induced a greater reduction in the mRNA levels of SP2 and KLF6 target genes compared to transfection with the pcDNA3.1-Ank_2 plasmids. For detailed real-time PCR results, refer to Figure 4.

**Figure 5:**
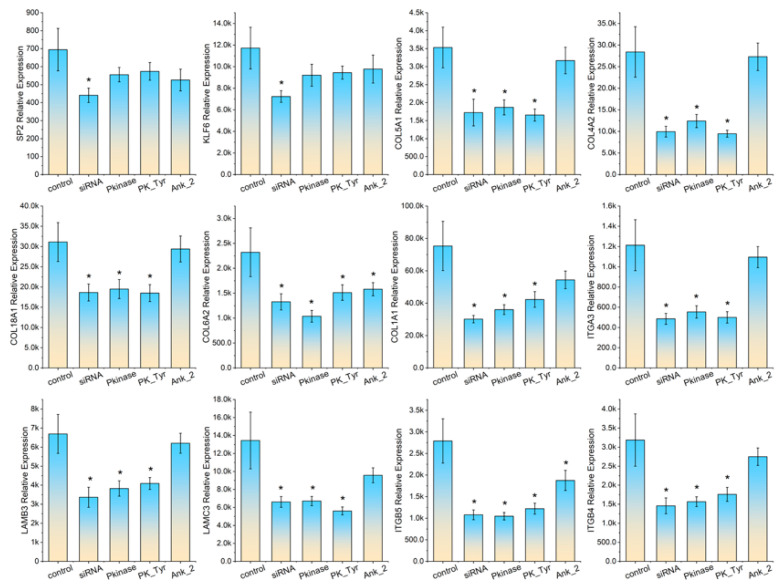
HeLa cells were transfected with SP2 siRNA, KLF6 siRNA, pcDNA3.1-Pkinase, pcDNA3.1-PK_Tyr, or pcDNA3.1-Ank_2 plasmids; the transcriptional levels of SP2, KLF6, and their target genes were subsequently analyzed. Bar heights represent relative expression normalized to GAPDH, and asterisks signify statistically significant differences (*p* < 0.05).

In cultured HeLa cells, the changes in transcription levels of the selected genes were similar with those observed in 3D4/21. SP2 and KLF6 siRNA treatment significantly reduced SP2 and KLF6 mRNA levels. There was no significance in SP2 and KLF6 transcription levels between cells transfected with pcDNA3.1-Pkinase, pcDNA3.1-PK_Tyr, or pcDNA3.1-Ank_2 plasmids and the control group. SP2 siRNA treatment significantly reduced the transcription levels of its target genes COL5A1, COL4A2, COL18A1, COL6A2, and COL1A1; KLF6 siRNA treatment significantly reduced the transcription levels of its target genes ITGA3, LAMB3, LAMC3, ITGB5, and ITGB4 in HeLa cells. Transfection of HeLa cells with pcDNA3.1-Pkinase and pcDNA3.1-PK_Tyr plasmids significantly reduced the transcription levels of COL5A1, COL4A2, COL18A1, COL6A2, COL1A1, ITGA3, LAMB3, LAMC3, ITGB5, and ITGB4 compared to the control samples. Among the target genes of SP2, only COL6A2 and COL1A1 showed significant downregulation, while among the target genes of KLF6, only ITGB5 and LAMC3 were significantly downregulated in HeLa cells transfected with the pcDNA3.1-Ank_2 plasmids. Results from the HeLa cells further demonstrated that the pcDNA3.1-Pkinase and pcDNA3.1-PK_Tyr plasmids exhibited slightly stronger inhibitory effects on the before mentioned target genes compared to the pcDNA3.1-Ank_2 plasmids. For detailed real-time PCR results, refer to Figure 5.

## 4. Discussion

Cell adhesion represents a critical interface in virus–host interactions, serving as both a key pathway for ASFV disguise, invasion, and pathogenesis, and the molecular basis for host recognition and defense against ASFV. This study reveals an important metabolic hijacking strategy employed by ASFV against host cells. Our data indicate that ASFV suppresses the transcription of key genes in the cell adhesion pathway—ITGA3, LAMB3, LAMC3, ITGB5, and ITGB4—by targeting the SP2 transcription factor. These findings directly address our research hypothesis that ASFV manipulates host cell adhesion and collagen formation pathways through viral protein–host transcription factor interactions, providing mechanistic evidence for how ASFV proteins R238L and R298L reprogram host gene expression to facilitate infection.

It has been demonstrated that ITGA3 typically binds to the integrin β1 subunit to form α3β1 integrin heterodimers; its mediated stable adhesion serves as a physical barrier for the host against viral infection [31]. LAMB3 (laminin β3 subunit) is a key component of laminin, serving as the initial barrier and interface encountered by viruses upon cell contact and potentially acting as a critical viral binding site [32]. LAMC3 is another distinct laminin subunit that transmits signals via integrins (e.g., α3β1, α6β1, αvβ3) to regulate cell migration, polarization, and survival [33]. The coordinated downregulation of these laminin components suggests that ASFV systematically dismantles the extracellular matrix barrier, potentially facilitating viral spread and tissue invasion. ITGB5 (integrin β5) and ITGB4 (integrin β4) also play vital roles in cell adhesion and viral infection. ITGB5 functions as a co-receptor or endocytosis trigger for multiple viral entry pathways [34], while ITGB4 serves as an anchoring cornerstone and signaling hub in epithelial cells, contributing to antiviral defense mechanisms [35]. Previous studies have reported that the focal adhesion pathway, particularly integrins, is involved in regulating the entry, endocytosis, and infection of multiple viruses [2,3,4,19]. The simultaneous downregulation of both integrin subunits and their ligands indicates that ASFV employs a multi-pronged strategy to compromise cell adhesion at multiple levels. In this study, ITGA3, LAMB3, LAMC3, ITGB5, and ITGB4 exhibited downregulated expression trends in animal lung tissues and cells transfected with plasmids expressing Pkinase, PK_Tyr, and Ank_2 domains. This suggests that ASFV’s R238L and R298L proteins may disrupt the cellular adhesion barrier to promote infection, potentially by targeting the transcription factor SP2. Targeting SP2 could, on one hand, suppress the transcription of ITGA3, LAMB3, and LAMC2 to potentially aid initial viral capture. On the other hand, it could inhibit ITGB5 and ITGB4 to modulate endocytic signaling, with these effects collectively driving viral entry. This dual mechanism highlights the efficiency of ASFV’s host manipulation strategy, simultaneously targeting structural and signaling components of cell adhesion.

Additionally, the findings of this study indicate that ASFV’s R238L and R298L proteins target transcription factor KLF6 to suppress the transcription of collagen formation pathway genes COL5A1, COL4A2, COL18A1, COL6A2, and COL1A1, thereby inducing a collagen-downregulated state. COL5A1 copolymerizes with type I collagen, embedding itself on the surface of type I collagen fibers [36], while in type V collagen it exists as a heterotrimeric fiber [37]. Recent research demonstrated that COL6A2 is a major component of type VI collagen, serving as a connective anchor and signaling hub between cells and the matrix [38]. COL4A2 is a major component of type IV collagen, providing mechanical support and elasticity to the basement membrane while serving as a scaffold for other basement membrane components [39]. COL18A1, the α1 chain of type XVIII collagen, is a crucial basement membrane component. It functions as a vital physical barrier component and regulates angiogenesis and the extracellular matrix environment [40]. COL1A1, which encodes the α1 chain of type I collagen, serves as a marker of lung myofibroblast activation and fibrosis post SARS-CoV-2 infection [41]. The breadth of collagen types affected—from basement membrane collagens to fibrillar collagens—suggests that ASFV infection broadly compromises extracellular matrix integrity, which may explain the widespread tissue damage and hemorrhage observed in infected animals.

The most important features of ASF—acute high fever and severe organ hemorrhage—suggest that host tissues sustain damage to the integrity of collagen structures. Specifically, injury to vascular endothelial cells and the disruption of vascular wall integrity, which relies heavily on type IV collagen in the basement membrane and types I and III collagen in the supporting framework, can lead to hemorrhage when collagen synthesis is disrupted or its degradation is accelerated [42]. In chronic or subacute cases, tissue damage triggers a repair process characterized by excessive collagen deposition (fibrosis) [43]. Although no evidence indicates that ASFV possesses a direct switch targeting collagen synthesis, infection likely induces a state where impaired collagen synthesis coexists with accelerated degradation through mechanisms such as resource competition and transcriptional stress. This study identifies a specific mechanism—ASFV R238L and R298L proteins targeting KLF6—that contributes to this collagen disruption, providing a molecular link between viral infection and the pathological features of ASF. This constitutes the structural basis for induced hemorrhage and tissue damage, while also initiating subsequent fibrotic repair. The ASFV’s R238L and R298L proteins act on the host’s KLF6 protein to inhibit transcription of genes in the collagen degradation pathway. This presents one mechanism by which ASFV causes pathological tissue hemorrhage through systemic disruption of host cell homeostasis.

The transcriptional suppression of the aforementioned genes not only elucidates the regulation of host TFs SP2 and KLF6 by ASFV but also offers a new perspective on the mechanism by which ASFV modulates and adapts to the host cellular environment to achieve immune evasion through regulating the host cell’s collagen degradation signaling pathway and fecal adhesion signaling pathway. Furthermore, it opens a new dimension for ASFV treatment, shifting from merely suppressing viral replication to restoring host cellular homeostasis.

Several limitations should be acknowledged when interpreting these findings. First, while our transcriptional data suggest potential disruption of collagen formation and cell adhesion pathways, direct functional validation—such as assays assessing extracellular matrix deposition, collagen staining, or cell adhesion and barrier function—was not performed. Therefore, conclusions regarding tissue-level structural dysfunction, including collagen matrix loss or altered cell adhesion, are inferred from gene expression patterns rather than direct functional measurements. Second, although our FRET flow cytometry assays confirmed close-range interactions between specific domains of ASFV proteins (R238L/R298L) and host transcription factors (SP2/KLF6), these interactions were validated using truncated domains rather than full-length proteins and under overexpression conditions. Whether such interactions occur in the context of full-length viral proteins during natural infection, and whether they lead to the same transcriptional outcomes, remain to be investigated. Third, these interactions have not been confirmed in vivo using ASFV-infected pig lung tissue. Future studies incorporating functional assays, protein-level validation, and in vivo infection models will be necessary to fully confirm the proposed mechanisms and their pathological consequences.

## 5. Conclusions

This study reveals a novel mechanism by which African swine fever virus (ASFV) manipulates host cellular environments through targeting transcription factors SP2 and KLF6. Our findings demonstrate that ASFV proteins R238L and R298L bind to the zf-C2H2 domains of these transcription factors, leading to the suppression of genes involved in collagen formation and cell adhesion pathways. This transcriptional reprogramming likely compromises extracellular matrix integrity and cellular barrier function, contributing to the hemorrhage and tissue damage characteristics of ASFV infection. These insights not only advance our understanding of ASFV pathogenesis but also identify potential therapeutic targets for restoring host cellular homeostasis. Future studies should focus on validating these interactions in vivo using natural ASFV infection models and investigating whether restoring SP2 and KLF6 activity can ameliorate disease symptoms.

## Figures and Tables

**Table 1:** Top 30 differentially expressed genes between high and low p72-antibody animals in lung tissue.

Gene	Average (log2)	Fold Change (log2)	*p*-Value	*p*-Adjust	Description
CTSL	10.09	4.72	5.83 × 10^−33^	1.04 × 10^−28^	cathepsin L
SDC3	11.46	−3.69	3.49 × 10^−25^	3.10 × 10^−21^	syndecan 3
DYSF	10.93	−2.71	6.88 × 10^−22^	4.08 × 10^−18^	dysferlin
SPTBN2	8.64	−2.58	3.67 × 10^−16^	1.31 × 10^−12^	spectrin beta, non-erythrocytic 2
CCL2	9.08	2.71	4.82 × 10^−16^	1.43 × 10^−12^	chemokine (C-C motif) ligand 2
SPP1	9.59	3.25	1.48 × 10^−15^	3.76 × 10^−12^	secreted phosphoprotein 1
MARCO	12.51	−4.62	8.77 × 10^−15^	1.95 × 10^−11^	macrophage receptor with collagenous structure
VWF	12.42	−2.12	4.30 × 10^−14^	8.50 × 10^−11^	von Willebrand factor
PLVAP	11.85	−3.44	7.45 × 10^−14^	1.33 × 10^−10^	plasmalemma vesicle associated protein
CFP	10.55	−2.16	9.02 × 10^−14^	1.46 × 10^−10^	complement factor properdin
STXBP1	9.87	−2.20	2.54 × 10^−13^	3.48 × 10^−10^	syntaxin binding protein 1
NOTCH3	9.94	−1.95	4.01 × 10^−13^	5.10 × 10^−10^	notch receptor 3
DCHS1	9.51	−1.90	4.50 × 10^−13^	5.34 × 10^−10^	dachsous cadherin-related 1
AMBN	8.36	2.88	5.05 × 10^−13^	5.62 × 10^−10^	ameloblastin
MRC2	11.27	−1.81	7.37 × 10^−13^	7.72 × 10^−10^	mannose receptor C-type 2
IFI6	13.18	2.92	3.72 × 10^−12^	3.15 × 10^−9^	interferon alpha inducible protein 6
ADGRG1	10.39	−2.72	4.27 × 10^−12^	3.46 × 10^−9^	adhesion G protein-coupled receptor G1
TNS3	11.39	−1.95	5.09 × 10^−12^	3.94 × 10^−9^	tensin 3
CSF1R	12.01	−2.56	1.01 × 10^−11^	6.89 × 10^−9^	colony stimulating factor 1 receptor
COL4A6	9.20	−1.98	1.98 × 10^−11^	1.26 × 10^−8^	collagen type IV alpha 6 chain
FGD2	9.17	−1.98	2.29 × 10^−11^	1.40 × 10^−8^	FYVE, RhoGEF and PH domain containing 2
COL4A2	13.39	−2.57	2.69 × 10^−11^	1.55 × 10^−8^	collagen type IV alpha 2 chain
SLC7A11	7.20	2.02	3.42 × 10^−11^	1.90 × 10^−8^	solute carrier family 7 member 11
ATP8	5.98	3.31	4.28 × 10^−11^	2.31 × 10^−8^	ATP synthase F0 subunit 8
IFITM3	12.30	2.34	4.88 × 10^−11^	2.56 × 10^−8^	---
CXCL11	6.30	2.55	5.43 × 10^−11^	2.76 × 10^−8^	C-X-C motif chemokine ligand 11
CDH23	9.46	−1.77	5.86 × 10^−11^	2.90 × 10^−8^	---
SNORA73	9.29	2.50	9.18 × 10^−11^	4.20 × 10^−8^	small nucleolar RNA SNORA73 family
HSPG2	12.09	−1.84	1.21 × 10^−10^	5.37 × 10^−8^	heparan sulfate proteoglycan 2
SRGN	12.52	2.39	1.72 × 10^−10^	7.46 × 10^−8^	serglycin

## Data Availability

The original contributions presented in this study are included in the article/Supplementary Material. Further inquiries can be directed to the corresponding authors.

## Supplementary Materials

The following supporting information can be downloaded at: https://www.mdpi.com/article/10.3390/vetsci13030236/s1, Supplementary File S1: Supplementary Information; Supplementary File S2: Supplementary Figures; Supplementary Data S1: Differentially Expressed Genes; Supplementary Data S2: Significant Transcription Factors Identified by FLAVER Software Package.
