# Supplementary material for: African Swine Fever Virus R238L and R298L Disrupt Lung Cell Collagen Formation and Cell Adhesion Pathway by Targeting Transcription Factors Containing zf-C2H2 Domain
Source: Vet Sci. 2026 Feb 28;13(3):236. doi: 10.3390/vetsci13030236 (PMC13030306; doi:10.3390/vetsci13030236)
Supplement: Supplementary file 1 [file vetsci-13-00236-s001.zip › Supplemental File 1.pdf]

Supplemental Information

1. Sequence Domains for African Swine Fever Virus Proteins

> R298L_Pkinase

gaattcgcc**gccaccatgg**atGAGGGTGGCAGAAATAACGTCTTTTTGGGTCATCAAGTAGGGCAACC
CATTATATTTAAGTACGTTTCTAAAAAAGAAATTCCAGGAAATGAAGTCATCGTGTTGAAAGCCC
TGCAAGATACTCCCGGTGTTATTAAGCTTATCGAATATACCGAAAATGCTATGTATCATATACTA
ATCATAGAATATATTCCAAATAGTGTTGATCTACTTCATTATCATTACTTTAAAAAACTTGAGGAA
ACCGAAGCCAAAAAAATAATATTCCAGCTTATTCTTATTATACAAAACATTTATGAGAAGGGCTT
TATCCATGGGGATATTAAGGATGAGAACCTTATTATAGACATAAATCAAAAGATCATTAAGGTC
ATTGACTTTGGAAGCGCTGTTAGATTAGACGAAACCCGTCCCCAATATAATATGTTTGGAACATG
GGAATACGTGTGTCCAGAATTTTATTATTATGGTTATTACTACCAGCTTCCTTTAACCGTGTGGAC
GATAGGTATGGTCGCGGTTAATCTTTTTAGATTTCGTGCAGAAAATTTTTATTTAAATGATATCCT
GAAACGGGAAAATTATATTCCCGAGAACATTTCAGAGACGGGAAAACAGTTTATCACGGAATGT
TTAACAATTAATGAAAATAAGCGGCTTTCCTTTAAGAGTCTTGTATCACATCCTTGGTTT**GCTAGC**

> R298L_PK_Tyr

gaattcgccg**ccaccatgg**atAAGTACGTTTCTAAAAAAGAAATTCCAGGAAATGAAGTCATCGTGTT
GAAAGCCCTGCAAGATACTCCCGGTGTTATTAAGCTTATCGAATATACCGAAAATGCTATGTATC
ATATACTAATCATAGAATATATTCCAAATAGTGTTGATCTACTTCATTATCATTACTTTAAAAAACT
TGAGGAAACCGAAGCCAAAAAAATAATATTCCAGCTTATTCTTATTATACAAAACATTTATGAGA
AGGGCTTTATCCATGGGGATATTAAGGATGAGAACCTTATTATAGACATAAATCAAAAGATCATT
AAGGTCATTGACTTTGGAAGCGCTGTTAGATTAGACGAAACCCGTCCCCAATATAATATGTTTGG
AACATGGGAATACGTGTGTCCAGAATTTTATTATTATGGTTATTACTACCAGCTTCCTTTAACCGT
GTGGACGATAGGTATGGTCGCGGTTAATCTTTTTAGATTTCGTGCAGAAAATTTTTATTTAAATGA
TATCCTGAAACGGGAAAATTATATTCCCGAGAACATTTCAGAGACGGGAAAACAGTTTATCACG
GAATGTTTAACAATTAATGAAAATAAGCGGCTTTCCTTTAAGAGTCTTGTATCACAT**GCTAGC**

> R238L_Ank

gaattcgccg**ccaccatgg**atATTAAAAAACATATTAGAAATGGGAATCTTACACTATTTGAGGAATTT
TTTAAAACAGATCCGTGGATTGTCAATAGATGCGATAAAAATGGATCCTCGGTATTCATGTGGAT
ATGCATCTACGGACGTATAGACTTTTTAAAATTTCTTTTTGAACAAGAATCTTATCCTGGAGAAAT
AATTAACCCTCATAGGAGGGATAAAGATGGAAACTCTGCTTTACATTATTTAGCTGAGAAAAAA
AATCATTTAATCCTGGAAGGGGTGTTGGGCTATTTCGGAAAAAAT**GCTAGC**

2. Sequence Domains for Host's Transcription Factors

> SP2_zf-C2H2

gaattcgccg**ccaccatgg**atCTGCGGGCTCACGTGCGCCTGCACACCGGCGAGCGGCCCTTTGTCTG
CAACTGGTTCTTCTGTGGGAAGAGGTTCACCCGGAGTGACGAGCTCCAGCGACACGCCCGCAC
CCACACAGGGGACAAACGCTTCGAGTGCGCCCAGTGTCAGAAGCGCTTCATGAGGAGCGACC
ACCTCACCAAGCATTACAAGACCCAC**GCTAGC**

> KLF6_zf-C2H2

<u>gaattc</u>gccg**ccaccatgg**atGACAAGGGCAGCGGGACGCGTCCCCGGACGGCCGCAGAAGGGTG
CATCGGTGCCACTTTAACGGCTGCCGGAAAGTTTACACCAAAAGCTCCCACTTGAAAGCACATC
AGCGCACCCACACAGGAGAAAAGCCTTACAGATGCTCATGGGAAGGGTGTGAGTGGCGTTTTG
CAAGAAGCGATGAGTTAACCAGACACTTCAGAAAGCACACTGGTGCCAAGCCTTTTAAATGTTC
CCACTGTGACAGGTGTTTCTCCAGGTCCGACCACCTGGCCCTGCACATGAAGAGGCAC**<u>GCTAG</u>**
<u>C</u>

3. Tag Sequences

> SNAP-tag

<u>GCTAGC</u>atggacaaagattgcgaaatgaaacgtaccaccctggatagcccgctgggcaaactggaactgagcggctgcg
aacagggcctgcatgaaattaaactgctgggtaaaggcaccagcgcggccgatgcggttgaagttccggccccggccgccgt
gctgggtggtccggaaccgctgatgcaggcgaccgcgtggctgaacgcgtattttcatcagccggaagcgattgaagaatttc
cggttccggcgctgcatcatccggtgtttcagcaggagagctttacccgtcaggtgctgtggaaactgctgaaagtggttaaatt
tggcgaagtgattagctatcagcagctggcggccctggcgggtaatccggcggccaccgccgccgttaaaaccgcgctgagc
ggtaacccggtgccgattctgattccgtgccatcgtgtggttagctctagcggtgcggttggcggttatgaaggtggtctggcg
gtgaaagagtggctgctggcccatgaaggtcatcgtctgggtaaaccgggtctgggatga**<u>ctcgag</u>**

> CLIP-tag

<u>GCTAGC</u>atggacaaagactgcgaaatgaagcgcaccaccctggatagccctctgggcaagctggaactgtctgggtgcga
acagggcctgcacgagatcatcttcctgggcaaaggaacatctgccgccgacgccgtggaagtgcctgccccagccgccgtg
ctgggcggaccagagccactgatccaggccaccgcctggctcaacgcctactttcaccagcctgaggccatcgaggagttccc
tgtgccagccctgcaccacccagtgttccagcaggagagctttacccgccaggtgctgtggaaactgctgaaagtggtgaagtt
cggagaggtcatcagcgagagccacctggccgccctggtgggcaatcccgccgccaccgccgccgtgaacaccgccctgga
cggaaatcccgtgcccattctgatcccctgccaccgggtggtgcagggcgacagcgacgtggggccctacctgggcgggctc
gccgtgaaagagtggctgctggcccacgagggccacagactgggcaagcctgggctgggttga**<u>ctcgag</u>**

4. Real-time PCR Primers

Table S1. Real-time PCR Primers

Gene	Forward Primer	Reverse Primer
COL1A1	AGGGGCCAAGACAGAGCAGAAACC	GCGGCCGAGGGGGAAGAAGAAA
COL4A2	CCCTCGGCCGACACGCTCAA	CTCGGGCTGCACGGCTTCATCTGT
COL5A1	CCGCCAACCAGGACACCATCTACG	CCAGCGCGGCCCCTTCTTCC
COL6A2	GGACGGCATCACGGGCAACGAGA	GCGGAGGGCACTGGAGGGAGGATG
COL18A1	GCGCCGCCGTGCCCATCGTTA	GGTCCGTCCGTCCGTCCGTCCAT
GAPDH	AGGCCGGGGCTCACTTG	CTCCAGGCGGCAGGTCAGAT
ITGA3	TGGTCGGCGCCCCCTATTACTTTG	AGAGCGCAGGGTCCAGCACAGATG
ITGB4	CAGGGCGGCGAGGACTACGAGAGC	CCCAGGGCCCAGGACAGCGGAAGC
ITGB5	AGCGACCGGGGGCACTGT	CGGGCTCTGGATCGCTCACTCT
KLF6	GGCGTTTTGCAAGAAGCGATGAG	TACAGCCCACCCAACCACTACCA
LAMB3	GGCCTGCCACCCCTGCTTCC	CCACCGGCGCCTGCTTCTCG

LAMC3	CGCCACCACAACGCCTCCTACCTC	TGCAACACGGGGCTCTCCTCAAAG
SP2	CGGCCCTCCCCTCTCACCACAA	GAAAAAGAGGGGGATCGGGAAGTC

5. siRNAs Used in This Study

SP2: AUUCCAAAGCUAUUCUUGCCA

KLF6: UGAGAAAACAGUUUCUAAGCU
