# Supplementary material for: African Swine Fever Virus R238L and R298L Disrupt Lung Cell Collagen Formation and Cell Adhesion Pathway by Targeting Transcription Factors Containing zf-C2H2 Domain
Source: Vet Sci. 2026 Feb 28;13(3):236. doi: 10.3390/vetsci13030236 (PMC13030306; doi:10.3390/vetsci13030236)
Supplement: Supplementary file 1 [file vetsci-13-00236-s001.zip › Supplemental File 2.pdf]

Supplementary Figures

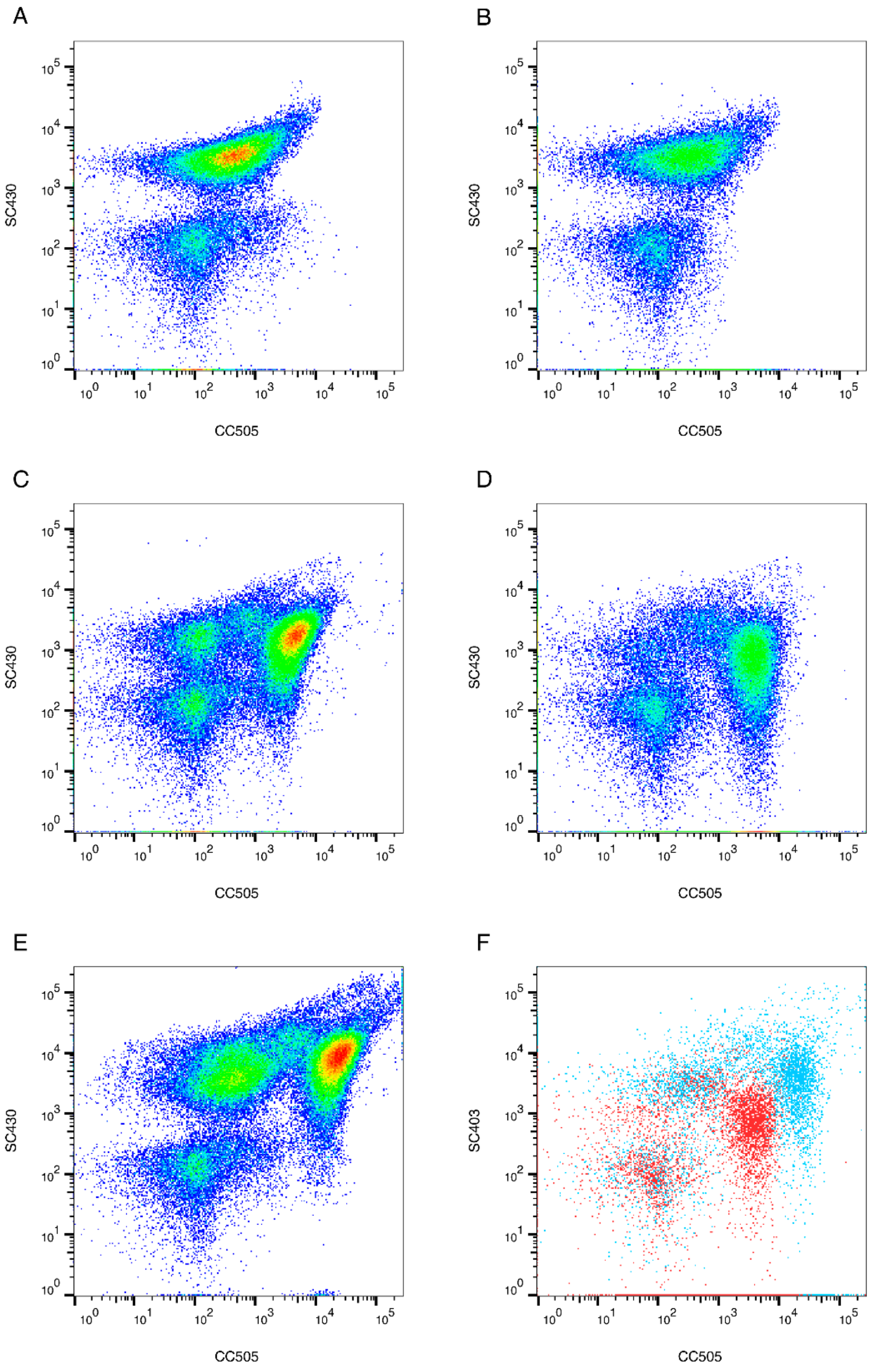

Figure S1. A: Uncompensated SC430 Only Samples. B: Compensated SC430 Only Samples. C: Uncompensated CC505 Only Samples. D: Compensated CC505 Only Samples. E: Uncompensated Field FRET Samples. F: Compensated Field FRET Samples (Blue) Overlapped with compensated CC505 Only Samples (Red).
